# Implementation of a Follow-Up System for Pediatric Sepsis Survivors in a Large Academic Pediatric Intensive Care Unit

**DOI:** 10.3389/fped.2021.691692

**Published:** 2021-06-04

**Authors:** Julie C. Fitzgerald, Nancy-Ann Kelly, Christopher Hickey, Fran Balamuth, Nina H. Thomas, Annique Hogan, Noelle J. Stack, Tara Trimarchi, Scott L. Weiss

**Affiliations:** ^1^Department of Anesthesiology and Critical Care Medicine, Children's Hospital of Philadelphia, Philadelphia, PA, United States; ^2^Department of Anesthesiology, Perelman School of Medicine, The University of Pennsylvania, Philadelphia, PA, United States; ^3^Pediatric Sepsis Program, Children's Hospital of Philadelphia, Philadelphia, PA, United States; ^4^Department of Pediatrics, Division of Emergency Medicine, Children's Hospital of Philadelphia, Philadelphia, PA, United States; ^5^Department of Pediatrics, Perelman School of Medicine, The University of Pennsylvania, Philadelphia, PA, United States; ^6^Department of Child and Adolescent Psychiatry and Behavioral Sciences, Children's Hospital of Philadelphia, Philadelphia, PA, United States; ^7^Department of Psychiatry, Perelman School of Medicine, The University of Pennsylvania, Philadelphia, PA, United States; ^8^Department of Pediatrics, Division of General Pediatrics, Children's Hospital of Philadelphia, Philadelphia, PA, United States; ^9^Care Management Department, Children's Hospital of Philadelphia, Philadelphia, PA, United States

**Keywords:** sepsis, child, pediatric intensive care, follow-up, survivor

## Abstract

**Background:** Survivors of pediatric sepsis often develop new morbidities and deterioration in quality of life after sepsis, leading to a need for improved follow-up for children who survive sepsis.

**Objective:** To implement a follow-up system for pediatric sepsis survivors in a pediatric health system.

**Methods:** We performed a retrospective case series of patients treated for sepsis from October 2018 through October 2019 in a pediatric intensive care unit in a quaternary children's hospital, and describe implementation of a follow-up system for sepsis survivors. Program planning started in 2017 with multidisciplinary meetings including physical, occupational, and speech therapists, teachers, neuropsychologists, and coordinators from other survivorship programs (neonatology, stroke, and oncology). In 2018, a workshop was held to consult with local and national experts. The Pediatric Sepsis Survivorship Program launched in October 2018 led by a nurse coordinator who met with families to educate about sepsis and offer post-discharge follow-up. Patients with high pre-existing medical complexity or established subspecialty care were referred for follow-up through existing care coordination or subspecialty services plus guidance to monitor for post-sepsis morbidity. For patients with low-moderate medical complexity, the nurse coordinator administered a telephone-based health-assessment 2–3 months after discharge to screen for new physical or psychosocial morbidity. Patients flagged with concerns were referred to their primary physician and/or to expedited neuropsychological evaluation to utilize existing medical services.

**Results:** Of 80 sepsis patients, 10 died, 20 were referred to care coordination by the program, and 13 had subspecialty follow-up. Five patients were followed in different health systems, four were adults not appropriate for existing follow-up programs, four remained hospitalized, and four were missed due to short stay or unavailable caregivers. The remaining 20 patients were scheduled for follow-up with the Pediatric Sepsis Program. Nine patients completed the telephone assessment. Four patients were receiving new physical or occupational therapy, and one patient was referred for neuropsychology evaluation due to new difficulties with attention, behavior, and completion of school tasks.

**Conclusions:** Implementation of an efficient, low-cost pediatric sepsis survivorship program was successful by utilizing existing systems of care, when available, and filling a follow-up gap in screening for select patients.

## Introduction

Sepsis contributes to significant morbidity and mortality in children (1–4). Over time, an increasing incidence of sepsis has been reported, with a suggestion of decreasing mortality (1–3). However, pediatric sepsis survivors are at risk for late mortality and readmission, as well as new disability (4–8). Over one-third of pediatric sepsis survivors have been reported to have new disability one to three months after sepsis diagnosis (4, 6), with physical effects and behavioral impacts lasting years in meningococcal sepsis (9, 10). In a multicenter, prospective study of health-related quality of life (HRQL) after community-acquired pediatric septic shock, over one-third of children surviving to hospital discharge had deterioration in HRQL on an in-depth assessment 1 year after hospital discharge (11). The largest decrease was seen in the first month after discharge with 50% of survivors below baseline HRQL, with some recovery that plateaued between 3 months and 1 year after discharge. Children with more organ dysfunction and those requiring higher levels of vasoactive infusion support were at highest risk of serious deterioration in HRQL (12). Furthermore, children without chronic comorbidities were disproportionately impacted by deterioration in HRQL (13).

Increasing recognition of new morbidities that impact quality of life for children who survive sepsis has led to calls to enhance follow-up after hospital discharge. Adult approaches to follow-up care after critical illness often involve clinics staffed by intensivists who provide outpatient care as part of their clinical repertoire (14). However, pediatric emergency and critical care clinicians lack training and resources for outpatient follow-up and most primary care and subspeciality follow-up appointments are scheduled too soon after hospital discharge to effectively screen for lingering morbidity. Primary care physicians and subspecialists may not be aware of the risk for lingering morbidity after sepsis. Finally, families are often unaware and uneducated about symptoms of Post-Intensive Care Syndrome in pediatrics (PICS-p) (15). As a result, they may not connect new concerns with a recent illness or seek care despite new morbidity after hospital discharge. Thus, there is an unmet need to implement an efficient approach to identify post-sepsis morbidity and to provide appropriate follow-up to ensure new health issues are managed after sepsis.

We have established a Pediatric Sepsis Program to serve as a central home for all sepsis-related research, clinical, and quality improvement activities at our institution. An initial goal of the program was to develop a survivorship program for children who are cared for in our pediatric intensive care unit (PICU) with sepsis. We aimed to develop a system that would empower families to better anticipate and identify post-sepsis morbidity and provide flexible, efficient follow-up for a heterogeneous patient population that could address new physical and psychosocial needs several months after hospital discharge. We report the planning, development, and results of the first year of this unique system.

## Materials and Methods

### Setting

The Children's Hospital of Philadelphia (CHOP) Institutional Review Board (IRB) determined that reporting the development and implementation of this follow-up system meets the exemption criteria for IRB oversight per 45 CFR 46.104(d) 4(iii). This clinical program was developed as part of a Department of Pediatrics Chair's Initiative to create a comprehensive Pediatric Sepsis Program, and was designed in a large, quaternary care medical-surgical PICU with 60 beds averaging over 4,000 annual PICU admissions.

### Planning

A multidisciplinary group including PICU and Physical Medicine & Rehabilitation physicians; physical, occupational, and speech therapists; a hospital-school liaison; a family advocate; a neuropsychologist; and program coordinators from other established acute care follow-up programs in the institution (e.g., neonatology, stroke, and oncology) was assembled to discuss the potential follow-up needs of sepsis survivors, avenues of follow-up currently available to sepsis survivors, and structures of other follow-up programs. The group sketched out different potential designs for a follow-up system for sepsis survivors at CHOP, including a possible dedicated sepsis follow-up clinic. The group developed data collection tools to help identify the post-discharge needs of sepsis survivors cared for at CHOP to help customize the follow-up system.

#### Needs Assessment

With the input of the neuropsychologist, a questionnaire was developed using REDCap electronic data capture tools hosted at CHOP to help the team understand new post-discharge needs of sepsis survivors (Supplementary Figure 1). Between October 2017, and March 2018, parents or guardians of patients treated for sepsis in the CHOP PICU were approached to introduce the questionnaire and obtain contact information. Two to four months after hospital discharge, a link to the questionnaire was sent via email to parents of sepsis survivors, with up to two reminders sent every 1–2 weeks if not completed. Concurrent with this, the local CHOP Virtual PICU Systems, Inc., database (VPS) was queried to measure the change in Functional Status Scale (16, 17) scores for patients with severe sepsis or septic shock (18) treated in the CHOP PICU between April, 2017, and February, 2018. VPS is a pediatric critical care registry and data are collected by trained PICU nurses. Rigorous data quality control procedures, including quarterly interrater reliability testing, are used to ensure data quality and reliability. FSS scores are determined by the trained nurse abstractors using data in the medical record at the time of PICU admission and PICU discharge.

**Figure 1:**
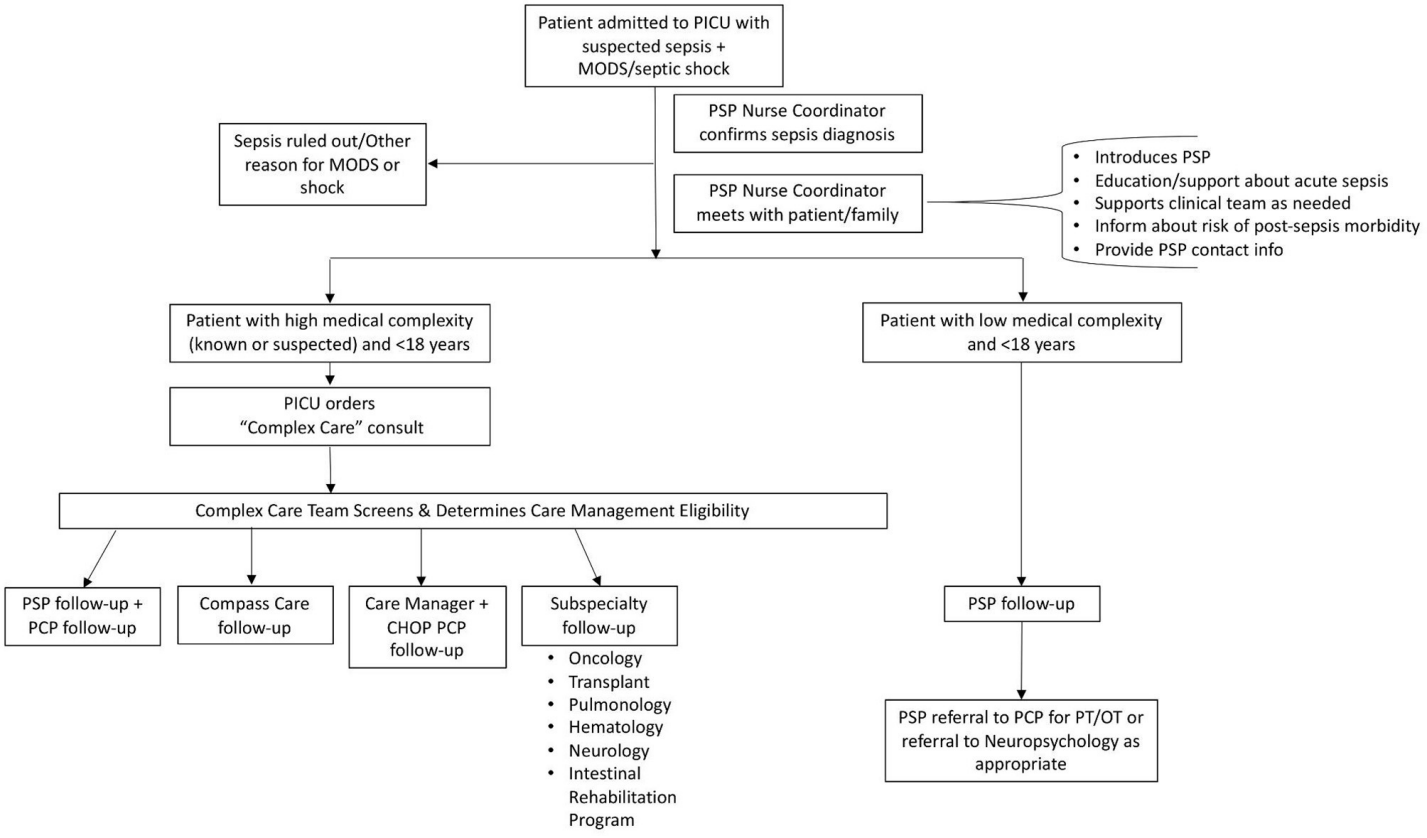
Initial sepsis survivorship workflow. The planning phase resulted in the initial follow-up workflow for patients cared for in the PICU with sepsis. PICU, Pediatric Intensive Care Unit; MODS, multiple organ dysfunction syndrome; PSP, Pediatric Sepsis Program; PCP, primary care provider; CHOP, Children's Hospital of Philadelphia; PT/OT, physical therapy/occupational therapy.

In June, 2018, a full day workshop was held to review the needs assessment and share insights from national experts in outcomes after pediatric sepsis and from local leaders of other CHOP follow-up programs. This workshop, along with ongoing guidance from hospital administration, helped inform a potential workflow for sepsis survivorship that ultimately did not involve establishing a new clinic. A care management team within CHOP was expanding scope concurrent with this work, and partnered with the sepsis team to build customized paths of follow-up for patients with varying complexity of care. The initial workflow design is presented in Figure 1.

### Implementation

A nurse coordinator with critical care experience was hired to contact clinical teams in the PICU to ascertain which patients in the PICU had septic shock or sepsis with multiple organ dysfunction (19), confirm the diagnosis with the Pediatric Sepsis Program physicians, make initial contact with and provide sepsis-related education to families on both acute aspects of sepsis and potential post-sepsis morbidity (both verbally and through a customized brochure), determine appropriate follow-up plan, and then recommend consultation to the appropriate specialty service or schedule post-discharge telephone follow-up through the CHOP Pediatric Sepsis Program. Screening of current PICU patients was performed two to three times per week by the nurse coordinator.

Previously healthy children and those with few pre-existing complex health conditions were identified for post-discharge follow-up through the CHOP Pediatric Sepsis Program. The nurse coordinator offered the family a post-discharge telephone-based health assessment, set a date and time for the follow-up with the family (~2–3 months after hospital discharge), obtained contact information, then sent a reminder letter via email followed by text messages to the family as the follow-up date approached. The follow-up assessment was developed with help from the multidisciplinary planning team (Supplementary Figure 2), and administered over the telephone to the parent or guardian of the sepsis survivor by the nurse coordinator, with the results recorded in a REDCap database. For adolescent patients, self-reported data was also collected from the patient when possible. Patients with new motor deficits noted on the questionnaire were referred to their primary care physician for physical, occupational, or speech therapy prescriptions, as appropriate. Patients with new psychosocial or educational/cognitive needs were scheduled for expedited neuropsychological evaluation. For patients whose primary care physician was within the CHOP system, contact was also made with the practice's Care Manager to assist with new needs and ensuring recommended follow-up is complete. We also developed a letter that a parent or guardian could give to a teacher or coach that provided general education about potential challenges facing children who survive sepsis.

**Figure 2:**
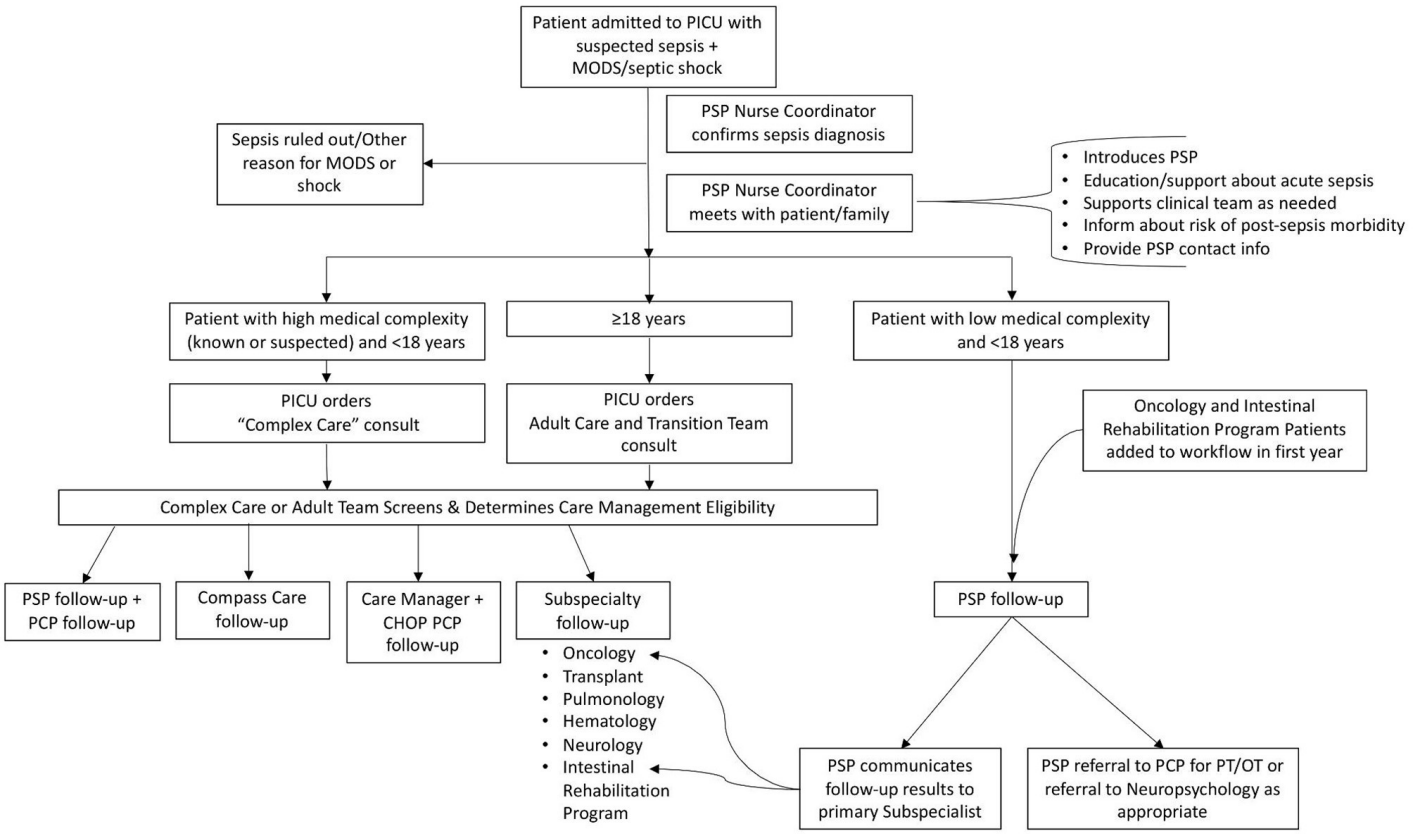
Updated sepsis survivorship workflow. The follow-up workflow was updated during the first year of the program. PICU, Pediatric Intensive Care Unit; MODS, multiple organ dysfunction syndrome; PSP, Pediatric Sepsis Program; PCP, primary care provider; CHOP, Children's Hospital of Philadelphia; PT/OT, physical therapy/occupational therapy.

Children and adults with specific comorbidities (e.g., congenital heart disease, malignancy) or those referred in through the Global Patient Services program (patients who traveled to the United States for specific subspecialty care for complex conditions), who were receiving frequent, close follow-up with a specialty service were followed as usual by their specialists. However, during the initial year of the program, in discussion with the Oncology and Intestinal Rehabilitation Program services, it was jointly determined that the sepsis follow-up may not fully overlap with normal Oncology and Intestinal Rehabilitation Program follow-up. The workflow was amended to include patients with malignancy and chronic intestinal dysfunction in the CHOP Pediatric Sepsis Program telephone follow-up program, with the plan to review the results of the follow-up with the respective subspecialty providers (Figure 2, updated workflow).

Children with a high degree of pre-existing medical complexity and/or significant technology dependence were referred to the inpatient complex care team. The sepsis nurse coordinator assisted the PICU providers in placing a consult and contacting this team about the patient during the inpatient stay. The complex care team met the patient and then helped determine if outpatient follow-up should be conducted with the analogous outpatient complex care team, with the primary care physician and associated Care Manager, or with an already involved specialty service.

Adult patients are typically only cared for in the CHOP PICU if an established relationship with a CHOP specialty service exists, so initially these patients were scheduled for routine follow-up with their specialty provider. Near the end of the first year, an adult medical team that assisted patients over 18 years with transition to adult care (the Adult Care and Transition Team) broadened their scope to function more similarly to the pediatric complex care team. The sepsis follow-up workflow was amended (Figure 2) to include a consult to this adult team for adult sepsis patients during the inpatient hospital stay, with outpatient follow-up with this team scheduled after discharge.

The sepsis nurse coordinator (NK) and Pediatric Sepsis Program directors (JCF, FB, SLW) met every 2 weeks to review new patients identified, plans for follow-up, and, for those patients scheduled for telephone follow-up, status of pending and completed follow-ups.

### Implementation Outcome

All patients identified by the sepsis nurse coordinator for1 year after the launch of the sepsis program follow-up (October 26, 2018—October 15, 2019) with septic shock, sepsis with multiple organ dysfunction, or pneumonia requiring mechanical ventilation (single organ dysfunction) had demographic information extracted, including age, sex, comorbidities, vital status at hospital discharge, and follow-up plan. For those patients scheduled for CHOP Pediatric Sepsis Program telephone follow-up, completion of scheduled follow-up, challenges related to completing follow-up, and results of telephone follow-up were recorded.

## Results

### Needs Assessment—Parental Input

During the needs assessment phase, email questionnaires were sent to parents or guardians of 28 sepsis survivors, and 12 responses were received. Eight of 12 respondents (67%) reported that their child received physical and/or occupational therapy during the inpatient hospitalization. One (8%) reported their child had acute inpatient rehabilitation after the hospitalization. None reported new use of outpatient rehabilitation services, new Individualized Education Programs, or identified teacher or physician concerns about their child after discharge. However, three parents reported their child experienced at least one new morbidity after discharge. Examples of new morbidities included difficulty with endurance (walking, climbing stairs), emotional problems (anger, tantrums, impulsivity, and anxiety), and social concerns (playing with friends).

### Needs Assessment—Historical PICU Data

A VPS query of PICU admissions to our institution between April, 2017, and February, 2018, resulted in 168 cases of severe sepsis or septic shock, 135 (80%) of whom survived to discharge. Of the 135 survivors, 34 (25%) experienced a change in FSS of ≥3 indicating significant new morbidity. Thirty-six survivors (26%) were discharged to acute inpatient rehabilitation or a skilled nursing facility, including 18 of the 34 with new morbidity. However, 16 of 34 patients with increased FSS (47%) were discharged to home, suggesting a need to continue follow-up post-hospitalization that may not otherwise be addressed through referral to inpatient rehabilitation or a skilled nursing facility. Based on the needs assessment phase which indicated that many children had poorer functional status and new morbidities after surviving sepsis, we implemented a sepsis survivorship program, as described in the methods. This program started on October 26, 2018.

### Program Implementation

In the first year of the sepsis survivorship clinical program, 80 patients were identified by the nurse coordinator as being treated for sepsis in the PICU. Characteristics of the patients are described in Table 1. Only 20% of the patients had no comorbidities prior to sepsis, and 85% met criteria for septic shock. Ten patients (13%) died prior to follow-up. Seven patients (8.8%) were discharged to acute inpatient rehabilitation hospitals. Twenty patients were referred to complex care teams or care coordination services at their primary care office, and 13 patients had frequent, close subspecialty follow-up. Five patients had medical homes in separate health systems. When possible, the Pediatric Sepsis Program Directors reached out to the primary care providers in other health systems and to subspecialist physicians to provide anticipatory guidance after the sepsis hospitalization. Four patients were adult patients prior to our partnership with the expanding adult complex care team and were therefore not followed. Four patients remained hospitalized. Four additional patients were identified as appropriate for telephone assessment; however, two had short hospitalizations limiting the ability for initial contact with the nurse coordinator, and the parents or guardians of the other two patients were not able to be reached. Twenty patients with no medical history or low-moderate pre-existing medical complexity were identified for follow-up through the Pediatric Sepsis Program.

**Table 1:** Characteristics of patients identified for potential sepsis follow-up.

**Characteristic**	***n* = 80**
Age in years, median (IQR)	8 (2–16)
Male sex	51 (64%)
≥1 comorbid condition	64 (80%)
Septic shock	68 (85%)
Severe sepsis	6 (7.5%)
Pneumonia requiring mechanical ventilation	6 (7.5%)
Sepsis source	
Respiratory	41 (51%)
Bloodstream	11 (14%)
Abdomen	11 (14%)
Skin/soft tissue/bone	4 (5%)
Central nervous system	4 (5%)
Genitourinary	2 (3%)
Culture negative sepsis, unknown source	7 (9%)
Required invasive mechanical ventilation	68 (85%)
Required vasoactive infusion for shock	62 (78%)
Died prior to follow-up	10 (13%)
Follow-up plan	
Pediatric sepsis program telephone follow-up	20
Referred to pediatric or adult complex care team or care coordination team	20
Subspecialty follow-up	13
Followed in separate health system	5
Adult patient (prior to adult complex care team partnership)	4
Remain hospitalized	4
Missed (discharged prior to sepsis program contact or caregiver unavailable)	4

Of the patients referred to complex care teams, care coordination services at their primary care office, or to existing subspecialty follow-up, 52% completed the recommended follow-up and 9% transitioned care to other health systems. The remaining 39% who did not complete the recommended follow-up were patients referred to complex care teams, all of whom continued to have close follow-up with other existing subspecialists and were receiving outpatient rehabilitation therapeutic services.

The Pediatric Sepsis Program nurse coordinator made contact with the parent or guardian of all 20 patients identified for follow-up through the Pediatric Sepsis Program and provided education and set an appointment for follow-up. The telephone follow-up health assessment was completed for nine patients at a median of 85 (range 59 and 210) days after sepsis onset. The remaining 11 were not able to be contacted after multiple attempts by telephone, text message, and email, or declined further follow-up when contacted. Characteristics of patients completing the telephone follow-up assessment compared to those who did not complete the follow-up are shown in Table 2. While statistical analysis was not performed due to the sample size, the proportions of patients with Hispanic ethnicity and with public insurance were higher in the group who did not complete the telephone assessment. Nearly all patients were English-speaking, although an interpreter was available and used when contacting patients who were not primarily English-speaking.

**Table 2:** Characteristics of patients who did and did not complete Pediatric Sepsis Program telephone follow-up health assessment.

**Characteristic**	**Completed follow-up (*n* = 9)**	**Did not complete follow-up (*n* = 11)**
Age in years, median (IQR)	3 (2–9)	7 (3–16)
Male sex	8	6
Race		
White	3 (33%)	5 (45%)
Black	2 (22%)	3 (27%)
Asian	1 (11%)	0 (0%)
Other	3 (33%)	3 (27%)
Hispanic ethnicity	1 (11%)	4 (36%)
Primary language is English	9 (100%)	10 (91%)
Insurance		
Public	4 (44%)	7 (64%)
Private	5 (56%)	4 (36%)
≥1 comorbid condition	4 (44%)	6 (55%)
Sepsis source		
Respiratory	2 (22%)	5 (45%)
Bloodstream	0 (0%)	2 (18%)
Abdomen	2 (22%)	1 (9%)
Skin/soft tissue/bone	3 (33%)	0 (0%)
Central nervous system	1 (11%)	0 (0%)
Genitourinary	0 (0%)	1 (9%)
Culture negative sepsis, unknown source	1 (11%)	2 (18%)
Required invasive mechanical ventilation	7 (78%)	8 (73%)
Required vasoactive infusion	9 (100%)	8 (73%)
Hospital length of stay in days, median (IQR)	11 (8–21)	19 (16–27)
Discharged to acute inpatient rehabilitation after sepsis admission	2 (22%)	3 (27%)
Readmitted to acute care within 6 months of discharge	1 (11%)	2 (18%)

Five of nine patients completing telephone assessment had no pre-existing comorbid conditions prior to sepsis. New post-sepsis difficulties and needs were similar when comparing patients with and without comorbid conditions except more patients with comorbidities had returned to school and more patients without comorbidities were receiving new physical or occupational therapy (Table 3, statistical comparison not done due to small sample size). Of all nine patients completing the follow-up health assessment, six patients reported gross motor skills (walking, jumping, stair climbing) had not returned to baseline, five were not performing all activities they had performed pre-illness, and four had decreased endurance. Four of these nine patients were receiving physical or occupational therapies that they were not receiving pre-illness. Six patients had returned to school, of whom three had new individual education plans (IEPs) in place. The remaining three patients were infants or toddlers who were not yet in school. One patient had new challenges with attention, focus, behavior, and completion of school tasks (reading and math, with a new IEP in place), and was referred for expedited neuropsychological evaluation.

**Table 3:** Follow-up results in previously healthy patients vs. patients with comorbid conditions.

**Follow-up question**	**Patients with no prior comorbid conditions (*n* = 5)**	**Patients with prior comorbid conditions (*n* = 4)**
Returned to school	2	4
New Individual Education Plan	1	2
New difficulty with reading or math	1	0
Gross motor skills not at baseline	3	3
Fine motor skills not at baseline	2	2
Not performing all pre-illness activities	3	2
New shortness of breath	3	2
Decrease in endurance	2	2
Not sleeping through the night	2	2
New physical or occupational therapy	3	1
New difficulty with staying focused	1	0
New pain	1	0
Total patients with new health issues	4	4
Total patients receiving new health interventions for new issues	3	2

## Discussion

Through multidisciplinary collaboration, we planned and implemented a follow-up program for pediatric sepsis survivors that provided flexibility based on patient characteristics and needs, while leveraging existing outpatient resources to supplement a new follow-up program that sought to minimize additional burdens on families. During the implementation process, we built new partnerships and modified our workflow to accommodate follow-up for patient populations we found were not adequately provided for in our initial planning, such as adult patients. Successful follow-up through our telephone-based follow-up program was challenging, with fewer than 50% of patients attending their telephone follow-up appointment and completing the questionnaire. Despite these challenges, we found that many patients who were followed up had new difficulties impacting their quality of life, with over half reporting they were not participating in all pre-illness activities and had not regained baseline gross motor skills. However, many of the survivors were successfully receiving new outpatient therapies and/or had new accommodations in place at school.

The parental surveys and local historical data analysis performed during our planning phase highlighted a need to improve and ensure access to follow-up for all sepsis survivors, especially those who were not already identified for post-discharge rehabilitation services and did not have ongoing subspeciality care. The planning phase of our program development also provided a chance for multiple providers to contribute designing the program workflow, and to increase awareness of the burden of new morbidities in sepsis survivors to outpatient providers, enabling partnerships that enhanced follow-up for this vulnerable population. We found that leveraging existing outpatient follow-up programs for complex patients allowed us to efficiently focus our critical care team resources on those patients who did not have a high degree of existing medical complexity, and who may be at highest risk for undiagnosed and untreated deficits after sepsis (13). Furthermore, embedding the follow-up program within existing clinical structures and utilization of existing clinical resources was cost-effective, requiring the hiring of a part-time nurse coordinator as the only new expenditure. It was also more feasible to implement than creating a new post-intensive care follow-up clinic modeled after adult clinics, and potentially less burdensome to families who had many other follow-up appointments.

After implementation, we found that many patients in our program had new difficulties impacting their quality of life after sepsis, which is consistent with research studies reporting new morbidities and impacts on HRQL in children after sepsis (4–11, 20). Based on prior literature, we anticipated more difficulties in psychosocial domains rather than physical functioning (20); however, only one patient reported difficulties with attention, focus, and completion of school activities. Interestingly, half of the patients who had returned to school had new IEPs in place despite the low reported rate of school challenges. Rather than psychosocial difficulties, many of the patients reported issues with gross motor skills (running, jumping, and stair climbing), shortness of breath, and decreased endurance. Additionally, many were having sleep disturbances at night, which could contribute to the gross motor difficulties, attention, and school difficulties, on top of the deconditioning that occurs during critical illness. While some of the patients with these difficulties were receiving physical and occupational therapy, our program provided another method to screen for deficits so physical and occupational therapy could be ordered for those not receiving these therapies.

During implementation of the program, we found that we needed continued flexibility in our workflow as we found patient populations that were not sufficiently accounted for in our original workflow design. We also partnered with subspecialty teams to better understand what functional domains are addressed in their ongoing outpatient care. We discovered that some morbidities that sepsis survivors may experience are not fully screened for in routine outpatient subspecialty visits, and have now expanded our telephone follow-up program to include Oncology patients and patients with chronic intestinal dysfunction (“short gut”). For these patients, challenges highlighted in the telephone assessment are reported to the subspecialist/medical home. As our program grows, we will continue to expand this model to other subspecialty patients. Because the core focus of our survivorship program is to educate families about post-sepsis symptoms and to screen for potential new morbidity concerns while referring formal diagnosis and assessment to existing health care systems, we are able to adapt to new and changing patient needs in an efficient, cost-effective manner.

A large challenge we encountered was difficulty in connecting with families for follow-up after hospital discharge. Less than half of patients attended their scheduled telephone follow-up appointment despite sending reminders through a variety of communication modalities, reaching out after missed appointments, and attempting to reschedule. It is possible that social determinants of health impacted the follow-up success. Those with public insurance and those with Hispanic ethnicity had lower rates of follow-up. We used interpreter services available at the hospital when contacting families who were not primarily English-speaking to limit this impact of potential language barriers, however, this did not seem to be a large contributor to challenges with completing follow-up. We explored whether being readmitted to the hospital during the time period of scheduled follow-up could have impacted family availability for follow-up, however, there was a low frequency of readmission during this time period. After pediatric sepsis, over a quarter of families experience moderate to high caregiver distress for up to a year after the sepsis episode (21). This may also be an explanation for our lost-to-follow-up rate. One reason for choosing a telephone follow-up model was to decrease the burden of additional outpatient appointments on families, and it is possible that an in-person clinic model would have struggled even more with successful follow-up (22). Interestingly, as the second year of the program has progressed through a pandemic, we are now seeing higher rates of follow-up, which may be related to a higher rate of acceptance of “virtual” medical appointments, or that more time is spent at home making it easier to have successful telephone appointments. Our program also provides education about sepsis and the potential for post-sepsis morbidity to families while still in the hospital. This aspect may empower families to seek care from other established providers even if follow-up through our program is not completed. Another potential benefit of our program to patients and families is our partnership with several independent charitable organizations that offer financial help to sepsis survivors. Educating families about these resources has helped develop a trusting relationship between our team and the families, and may contribute to increasing successful follow-up.

Our study has several limitations. The workflow we designed and implemented uses resources and outpatient programs that may be unique to our institution, potentially limiting generalizability to other healthcare systems. However, the theme of partnering with existing outpatient programs and knowledge sharing to provide anticipatory guidance and education about the expected challenges sepsis survivors face to the providers in these programs could be generalizable. Completion of follow-up for patients scheduled to have telephone appointments with our Pediatric Sepsis Program was quite challenging. This was not an unexpected challenge with a new program, and while our follow-up rate was lower than that reported for in-person neonatal follow-up clinics (23), it was higher than follow-up rates reported in adult post-intensive care clinics (22). To address this, we broadened our methods of contact during our initial implementation phase to include a variety of communication methods to optimize our rate of successful follow-up. Purchasing a dedicated mobile phone for our program allowed for text messaging with families, and was often a successful method of achieving a response. Another limitation was that our screening method was not as robust for identifying hospitalized sepsis patients as our electronic surveillance methods. We identified approximately half as many sepsis patients to approach for follow-up as we evaluated in our needs assessment phase over a similar amount of time. One explanation is that patients who were receiving end of life care or for whom death was imminent were not identified, logged, and approached by our team. This is reflected in the lower mortality rate seen in our implementation period. There were likely other patients who were missed as our screening did not occur daily, and is an area to target for future growth for our program.

Another limitation is the use of FSS scores determined by medical record review in the planning phase, which identified an estimated rate of significant new morbidity in sepsis survivors of 25%. While the VPS data registry used to collect FSS scores has high quality data, accurate determination of FSS domains from medical record data can be challenging and may not provide an accurate assessment of new morbidity. However, eight patients who completed the follow-up telephone assessment were found to have new health issues after sepsis. Assuming that all 11 patients who did not complete the telephone assessment had no new morbidities, the rate of new morbidities in our cohort would be 40% (8/20), slightly higher than the rate estimated by the FSS scores. It is likely that some of the patients not completing follow-up did have new morbidities, making the rate of new morbidities higher than 40%, and higher than that estimated by the FSS scores, but our telephone assessment may also pick up more subtle changes that may not be comparable to new morbidity defined using an FSS score. Finally, because the data from the first year of telephone follow-up is based on a small number of patients, it may not accurately reflect the population averages of new morbidities after pediatric sepsis. However, our primary aim for this report was to review that implementation of a follow-up system for pediatric sepsis survivors rather than study the epidemiology of post-sepsis morbidity. Moreover, it is possible that patients who did not complete the follow-up may have not felt the need to attend their appointment because they had no ongoing problems; thus, we may overestimate the proportion of patients with new morbidities after sepsis. Conversely, these patients may be experiencing moderate or high caregiver distress, and may be at even higher risk for post-sepsis morbidities and difficulty accessing care. Caregiver distress was not systematically screened for in the follow-up, but may be an area to incorporate into follow-up in the future.

In the process of planning and implementing this follow-up program for sepsis survivors, we learned several key lessons. Persistence was key in developing a relationship with families. Often a parent or guardian was not at the bedside when the nurse coordinator first attempted contact with the family. Repeated visits to the bedside and telephone calls to make contact helped in the ability to provide sepsis education and schedule telephone follow-up assessments. The introduction of an appointment letter with the date and time of the scheduled telephone follow-up assessment provided a written reminder and seemed to lend an official nature to the follow-up, possibly increasing follow-up success. Following up email communications with telephone calls several days later also increased the chances of successfully contacting families. Finally, other primary care and specialist providers were welcoming of our efforts to raise awareness of the potential for new morbidities and new needs after sepsis and appreciated our involvement in the care of their patients.

For other institutions interested in creating similar programs, it may be difficult to solicit funding from hospital administration for a program that does not directly generate income. We found that performing the needs assessment, in particular demonstrating the high estimated rate of new morbidity in sepsis survivors in historical patients cared for at our hospital, was helpful in justifying the costs of the program. In the Life After Pediatric Sepsis Evaluation study, it was shown that changes in FSS or Pediatric Overall Performance Category score between days 1 and 7 were highly predictive of death or severe deterioration in HRQL 3 months after sepsis (12). Incorporating these scores as screening tools to identify those most likely to benefit from a sepsis follow-up program might further refine the appropriate target population and limit costs associated with a program like this. Utilization of existing clinical resources also helped us limit the primary expenditures to salary for a part-time nurse coordinator. Our hope is that programs like this will not only improve HRQL for children who survive sepsis, but will also reduce readmissions and provide increased patient and family satisfaction with the health care system.

## Conclusions

Although PICS-p after sepsis is increasingly reported in the literature, a gap exists in the availability of outpatient follow-up to screen for and address these needs. We describe one method of helping to close this gap by leveraging and augmenting existing outpatient resources and developing a new telephone follow-up system to screen for new needs and refer concerns to appropriate health care professionals. Further research is needed to understand the impact of programs like this, and how to continually improve follow-up programs and completion of follow-up.

## Data Availability Statement

The raw data supporting the conclusions of this article will be made available by the authors, without undue reservation.

## Ethics Statement

The studies involving human participants were reviewed and approved by Children's Hospital of Philadelphia Institutional Review Board. Written informed consent from the participants' legal guardian/next of kin was not required to participate in this study in accordance with the national legislation and the institutional requirements.

## Author Contributions

JF contributed to the conception and design of the work, the acquisition, analysis, and interpretation of data for the work, and drafting the work and revising it critically for important intellectual content. N-AK, CH, FB, and SW contributed to the conception and design of the work, the acquisition, analysis, and interpretation of data for the work, and revising it critically for important intellectual content. NT, AH, NS, and TT contributed to the conception and design of the work, and revising it critically for important intellectual content. All authors gave final approval of the version to be published and agree to be accountable for all aspects of the work in ensuring that questions related to the accuracy or integrity of any part of the work are appropriately investigated and resolved.

## Conflict of Interest

The authors declare that the research was conducted in the absence of any commercial or financial relationships that could be construed as a potential conflict of interest.
