# Supplementary material for: Implementation of a Follow-Up System for Pediatric Sepsis Survivors in a Large Academic Pediatric Intensive Care Unit
Source: Front Pediatr. 2021 Jun 4;9:691692. doi: 10.3389/fped.2021.691692 (PMC8212949; doi:10.3389/fped.2021.691692)
Supplement: Supplementary file 1 [file Data_Sheet_1.PDF]

Supplementary Material

1 Supplementary Figures

Supplementary Figure 1. Parent questionnaire administered by email during needs assessment phase.

**Part 1: Please report your experience during and after the hospital admission for sepsis**	
What is today's date?	______________________
On approximately what date was your child admitted to the hospital that included a course of sepsis (or suspected sepsis)?	______________________
1. What was your child's age when he/she was treated for sepsis?<br>(enter number only - specify if this number refers to years, months, or days in the following question)	______________________
Age provided in days, months, or years?	◯ days<br>◯ months<br>◯ years
2. What is your child's sex (gender)?	◯ male<br>◯ female
3. What is your child's race and ethnicity?	◯ Alaskan/Pacific Islander<br>◯ Asian<br>◯ Black Hispanic<br>◯ Black Non-Hispanic<br>◯ Indian<br>◯ Native American<br>◯ White Hispanic<br>◯ White Non-Hispanic<br>◯ Other<br>◯ Prefer not to answer

4. Had you noticed any abnormalities in the following before your child's sepsis?

	Yes	No	I am not sure
a. Cognitive and school function, including attention, learning, thinking, or communicating	◯	◯	◯
b. Health and physical function, including motor skills, sleeping, or eating	◯	◯	◯
c. Mood and behavior, including excessive sadness, anxiety, or anger	◯	◯	◯
d. Experience of pain, including ongoing pain symptoms not related to an injury	◯	◯	◯

5. Did you child experience any of the following during his/her hospitalization for sepsis:

	Yes	No	I am not sure
Need for a breathing tube (sometimes called "intubation")	◯	◯	◯
Seizures	◯	◯	◯
Meningitis or encephalitis (sometimes call "brain infection", "brain inflammation", or "brain swelling")	◯	◯	◯

6. Did your child see a physical therapist (PT) and/or occupational therapist (OT) at any time during his/her hospitalization that included sepsis?

◯ Yes
◯ No
◯ I am not sure

If YES, what were the recommendations for after hospital discharge?
For example: "Follow-up with outpatient PT" or "Balance still needs to be addressed"

____

7. Did your child see a speech-language specialist any time during his/her hospitalization that included sepsis? ?

◯ Yes
◯ No
◯ I am not sure

If YES, were any speech, language, or eating/swallowing recommendations provided for after hospital discharge?

____

8. Has your child participated in a rehabilitation program since being discharged from the hospital after his/her sepsis illness?	◯ Yes, inpatient rehabilitation for PT/OT (or other therapy) for at least one day<br>◯ Yes, outpatient rehabilitation for PT/OT (of other therapy) services for a least one day<br>◯ No<br>◯ I am not sure
9. Has your child received any NEW or INCREASED therapy services since his/her sepsis illness?<br>For example: PT needed now but not before sepsis or more frequent PT now than before sepsis	☐ Physical or occupational therapy<br>☐ Speech/language therapy<br>☐ Neuropsychology, psychiatry, or behavioral health specialist<br>☐ Other<br>☐ No new therapy services<br>☐ I am not sure<br>(Select all that apply)
10. Prior to sepsis, did your child have an "Individualized Education Plan (IEP)", 504 Plan, or any other special accommodations (such as shortened school day, reduced workload, support with physical activity, etc)?	◯ Yes<br>◯ No<br>◯ I am not sure
11. Does your child have a new or updated "Individualized Education Plan (IEP)", 504 Plan, or any other special accommodations such as shortened school day, reduced workload, support with physical activity, etc, that was not present before sepsis?	◯ Yes<br>◯ No<br>◯ I am not sure
12. Has your child returned to school (or daycare) since his/her hospitalization?	◯ Yes<br>◯ No<br>◯ I am not sure
13. Have you or your child's teacher or daycare provider noticed a change in your child's academic performance since his/her discharge from the hospital	◯ Yes<br>◯ No<br>◯ I am not sure
If Yes, please describe the change in academic performance:	________________________________
14. Have you or your child's teacher or daycare provider noticed a change in your child's health or physical activity since his/her discharge from the hospital?	◯ Yes<br>◯ No<br>◯ I am not sure
If Yes, please describe the change in health or physical activity:	________________________________
15. Have you or your child reported to a doctor or other health care professional any concerns about long-term health and general well-being that have been noticed after the sepsis episode?	◯ Yes<br>◯ No<br>◯ I am not sure

Part 2: Please rate your child's CURRENT ABILITIES compared to his/or own abilities before sepsis.

We realize that not all children have the same baseline abilities, due to their age or pre-existing medical conditions. Please rate your child's current abilities compared to his/her own baseline ability before the sepsis episode.

For each area, please indicate if your child's functioning is:

1. Much worse than before the sepsis episode
2. A little worse than before the sepsis episode
3. About the same as before the sepsis episode
4. A little better than before the sepsis episode
5. Much better than before the sepsis episode
6. Not applicable (only if child is too young to demonstrate this skill)

1. Compared to before sepsis, my child's current:

	Is MUCH WORSE than before the sepsis episode	Is a LITTLE WORSE than before the sepsis episode	is ABOUT THE SAME as before the sepsis episode	Is a LITTLE BETTER than before the sepsis episode	Is MUCH BETTER than before the sepsis episode	Is not applicable (only if child is too young to demonstrate this skill)
A. Ability to focus on activities at home	○	○	○	○	○	○
B. Ability to focus on activities at school	○	○	○	○	○	○
C. Ability to listen to and follow instructions	○	○	○	○	○	○
D. Ability to stay organized, such as cleaning up toys or organizing a backpack	○	○	○	○	○	○
E. Memory	○	○	○	○	○	○
F. Expressive language skills (such as word finding and ability to put thoughts into words)	○	○	○	○	○	○
G. Language articulation (such as speaking clearly and at a typical pace)	○	○	○	○	○	○
H. Reading words	○	○	○	○	○	○
I. Understanding what he/she is reading	○	○	○	○	○	○
J. Handwriting or writing words	○	○	○	○	○	○
K. Math skills	○	○	○	○	○	○
L. Vision	○	○	○	○	○	○
M. Hearing	○	○	○	○	○	○

2. Compared to before sepsis, my child's current:

	Is MUCH WORSE than before the sepsis episode	Is a LITTLE WORSE than before the sepsis episode	is ABOUT THE SAME as before the sepsis episode	Is a LITTLE BETTER than before the sepsis episode	Is MUCH BETTER than before the sepsis episode	Is not applicable (only if child is too young to demonstrate this skill)
A. Gross motor skills, such as walking, jumping, going upstairs and running	○	○	○	○	○	○
B. Fine motor skills, such as writing, coloring, or fastening buttons	○	○	○	○	○	○
C. Coordination, such as playing sports, riding a bike, or climbing a tree	○	○	○	○	○	○
D. Physical endurance, such as distance he/she can run before getting tired	○	○	○	○	○	○
E. Physical ability to do the activities that he/she likes	○	○	○	○	○	○
F. Ability to sleep through the night or naps	○	○	○	○	○	○
G. Appetite at mealtimes	○	○	○	○	○	○
H. Ability to feed him/herself	○	○	○	○	○	○
I. Ability to swallow food	○	○	○	○	○	○

3. Compared to before sepsis, my child's current:

	Is MUCH WORSE than before the sepsis episode	Is a LITTLE WORSE than before the sepsis episode	is ABOUT THE SAME as before the sepsis episode	Is a LITTLE BETTER than before the sepsis episode	Is MUCH BETTER than before the sepsis episode	Is not applicable (only if child is too young to demonstrate this skill)
A. Amount of time that my child is happy	○	○	○	○	○	○
B. Amount of time that my child is sad or depressed	○	○	○	○	○	○
C. Amount of time that my child is worried or anxious	○	○	○	○	○	○
D. Amount of time that my child is angry	○	○	○	○	○	○
E. Amount of time that my child is tired or fatigued	○	○	○	○	○	○
F. Amount of time that my child is hyperactive	○	○	○	○	○	○
G. Frequency or severity of behavioral outbursts, such as yelling or tantrums	○	○	○	○	○	○
H. Frequency or severity of impulse actions, such as grabbing or interrupting	○	○	○	○	○	○
I. Getting along well with friends or classmates	○	○	○	○	○	○

4. Compared to before sepsis, my child's current:

	Is MUCH WORSE than before the sepsis episode	Is a LITTLE WORSE than before the sepsis episode	is ABOUT THE SAME as before the sepsis episode	Is a LITTLE BETTER than before the sepsis episode	Is MUCH BETTER than before the sepsis episode	Is not applicable (only if child is too young to demonstrate this skill)
A. Amount of time in pain (not related to a new injury)	○	○	○	○	○	○
B. Severity of pain not related to a new injury	○	○	○	○	○	○
C. Concern about doctors	○	○	○	○	○	○
D. Anxiety about the hospital	○	○	○	○	○	○

5. Optional: Please describe any other concerns you have about your child's CURRENT health and well-being compared to before sepsis. ________________________________

Part 3: Please rate your child at the current time (that is, now that it is after the episode of sepsis) compared to OTHER CHILDREN THEIR OWN AGE

We realize that not all children have the same baseline abilities, due to their age or pre-existing medical conditions. Please rate your child's current abilities compared to other healthy children around his/her age even if the difference from other children may not have been related to sepsis (that is, the same difference would have been present before sepsis).

For each area, please indicate if your child's functioning is:

1. Much worse than others their age
2. A little worse than others their age
3. About the same as others their age
4. A little better than others their age
5. Much better than others their age
6. Not applicable (only if child is too young to demonstrate this skill)

1. Compared to OTHER CHILDREN HIS/HER AGE, my child's current:

	Is MUCH WORSE than other children his/her age	Is a LITTLE WORSE than other children his/her age	is ABOUT THE SAME as other children his/her age	Is a LITTLE BETTER than other children his/her age	Is MUCH BETTER than other children his/her age	Is not applicable (only if child is too young to demonstrate this skill)
A. Ability to focus on activities at home	○	○	○	○	○	○
B. Ability to focus on activities at school	○	○	○	○	○	○
C. Ability to listen to and follow instructions	○	○	○	○	○	○
D. Ability to stay organized, such as cleaning up toys or organizing a backpack	○	○	○	○	○	○
E. Memory	○	○	○	○	○	○
F. Expressive language skills (such as word finding and ability to put thoughts into words)	○	○	○	○	○	○
G. Language articulation (such as speaking clearly and at a typical pace)	○	○	○	○	○	○

2. Compared to OTHER CHILDREN HIS/HER AGE, my child's current:

	Is MUCH WORSE than other children his/her age	Is a LITTLE WORSE than other children his/her age	is ABOUT THE SAME as other children his/her age	Is a LITTLE BETTER than other children his/her age	Is MUCH BETTER than other children his/her age	Is not applicable (only if child is too young to demonstrate this skill)
A. Gross motor skills, such as walking, jumping, going upstairs and running	○	○	○	○	○	○
B. Fine motor skills, such as writing, coloring, or fastening buttons	○	○	○	○	○	○
C. Coordination, such as playing sports, riding a bike, or climbing a tree	○	○	○	○	○	○
D. Physical endurance, such as distance he/she can run before getting tired	○	○	○	○	○	○
E. Physical ability to do the activities that he/she likes	○	○	○	○	○	○
F. Ability to sleep through the night or naps	○	○	○	○	○	○
G. Appetite at mealtimes	○	○	○	○	○	○
H. Ability to feed him/herself	○	○	○	○	○	○
I. Ability to swallow food	○	○	○	○	○	○

3. Compared to OTHER CHILDREN HIS/HER AGE, the current:

	Is MUCH WORSE than other children his/her age	Is a LITTLE WORSE than other children his/her age	is ABOUT THE SAME as other children his/her age	Is a LITTLE BETTER than other children his/her age	Is MUCH BETTER than other children his/her age	Is not applicable (only if child is too young to demonstrate this skill)
A. Amount of time that my child is happy	○	○	○	○	○	○
B. Amount of time that my child is sad or depressed	○	○	○	○	○	○
C. Amount of time that my child is worried or anxious	○	○	○	○	○	○
D. Amount of time that my child is angry	○	○	○	○	○	○
E. Amount of time that my child is tired or fatigued	○	○	○	○	○	○
F. Amount of time that my child is hyperactive	○	○	○	○	○	○
G. Frequency or severity of behavioral outbursts, such as yelling or tantrums	○	○	○	○	○	○
H. Frequency or severity of impulse actions, such as grabbing or interrupting	○	○	○	○	○	○
I. Getting along well with friends or classmates	○	○	○	○	○	○

4. Compared to OTHER CHILDREN HIS/HER AGE, my child's current:

	Is MUCH WORSE than other children his/her age	Is a LITTLE WORSE than other children his/her age	is ABOUT THE SAME as other children his/her age	Is a LITTLE BETTER than other children his/her age	Is MUCH BETTER than other children his/her age	Is not applicable (only if child is too young to demonstrate this skill)
A. Amount of time in pain (not related to a new injury)	○	○	○	○	○	○
B. Severity of pain not related to a new injury	○	○	○	○	○	○
C. Concern about doctors	○	○	○	○	○	○
D. Anxiety about the hospital	○	○	○	○	○	○

5. Optional: Please describe any other concerns you have about your child's health and well-being at the current time compared to OTHER CHILDREN HIS/HER AGE. ________________________________

Supplementary Figure 2. Follow-up health assessment administered by telephone during implementation.

**HEALTH ASSESSMENT**	
Has your child followed up with the pediatrician or other physicians since discharge?	☐ YES<br>☐ NO<br>☐ UNKNOWN
Is your child receiving Physical or Occupational therapy?	☐ YES<br>☐ NO<br>☐ YES but same as before
How is his/her appetite at meal times?	○ Much better than before sepsis<br>○ Little better than before sepsis<br>○ About the same as before sepsis<br>○ Little worse than before sepsis<br>○ Much worse than before sepsis
Is He/She feeding himself?	○ Much better than before sepsis<br>○ Little better than before sepsis<br>○ About the same as before sepsis<br>○ Little worse than before sepsis<br>○ Much worse than before sepsis<br>(exclude infants and GT dependent patients)
Have you noticed any issues with swallowing food?	○ Much better than before sepsis<br>○ Little better than before sepsis<br>○ About the same as before sepsis<br>○ Little worse than before sepsis<br>○ Much worse than before sepsis

**EDUCATION (COGNITIVE AND SCHOOL FUNCTION)**	
Has your child returned to school/preschool/daycare?	○ YES<br>○ NO<br>○ N/A
If the answer is no,<br>Are they missing time because of illness or to go to the doctors or hospital?	☐ hospital<br>☐ doctor's appointments<br>☐ illness<br>☐ other
If your child has not returned to school/preschool/daycare, why not?	______________________________
Do you feel as if he or she is up to par with the rest of the class?<br>(do you feel they have caught up from time missed during illness?)	○ YES<br>○ NO<br>○ N/A
Does your child have a new IEP/504 Plan?<br>(Individualized Education Plan)	☐ YES<br>☐ NO<br>☐ N/A

If NO to above; Do you think your child would benefit from accommodations or services in school that hey are not currently receiving?	____________________________
any new or worsening issues with vision or hearing?	◯ Yes<br>◯ No
Have you noticed a change in his/her handwriting?	◯ Yes<br>◯ No
Does he /she require more time completing projects, reading, puzzles, etc?	◯ Much better than before sepsis<br>◯ Little better than before sepsis<br>◯ About the same as before sepsis<br>◯ Little worse than before sepsis<br>◯ Much worse than before sepsis<br>◯ N/A
Have you noticed any issues with them comprehending the meaning of sentences (spoken or read)?	◯ Much better than before sepsis<br>◯ Little better than before sepsis<br>◯ About the same as before sepsis<br>◯ Little worse than before sepsis<br>◯ Much worse than before sepsis<br>◯ N/A
Have you noticed any new or worsening difficulty with math or reading?	◯ Much better than before sepsis<br>◯ Little better than before sepsis<br>◯ About the same as before sepsis<br>◯ Little worse than before sepsis<br>◯ Much worse than before sepsis<br>◯ N/A
Have you noticed any issues with paying attention to school activities or play activities?	◯ Much better than before sepsis<br>◯ Little better than before sepsis<br>◯ About the same as before sepsis<br>◯ Little worse than before sepsis<br>◯ Much worse than before sepsis<br>◯ N/A
Have you noticed any issues with his/her speech? (such as difficulty finding words, talking at a slower pace, slurring words)	◯ Much better than before sepsis<br>◯ Little better than before sepsis<br>◯ About the same as before sepsis<br>◯ Little worse than before sepsis<br>◯ Much worse than before sepsis<br>◯ N/A
Have you noticed him/her forgetting things?	◯ Much better than before sepsis<br>◯ Little better than before sepsis<br>◯ About the same as before sepsis<br>◯ Little worse than before sepsis<br>◯ Much worse than before sepsis<br>◯ N/A
Have you noticed an issue with him/her staying on task? (distracted while cleaning up toys or doing homework)	◯ Much better than before sepsis<br>◯ Little better than before sepsis<br>◯ About the same as before sepsis<br>◯ Little worse than before sepsis<br>◯ Much worse than before sepsis<br>◯ N/A

Have you noticed a difficulty with him/her staying in school all day? (frequent nurse visits, incomplete work coming home, he/she stating that they didn't have enough time to finish task)	◯ Much better than before sepsis<br>◯ Little better than before sepsis<br>◯ About the same as before sepsis<br>◯ Little worse than before sepsis<br>◯ Much worse than before sepsis<br>◯ N/A
Is he/she having issues with staying organized? Examples are organizing back pack or putting clothes away.	◯ Much better than before sepsis<br>◯ Little better than before sepsis<br>◯ About the same as before sepsis<br>◯ Little worse than before sepsis<br>◯ Much worse than before sepsis<br>◯ N/A
**ACTIVITY (PHYSICAL FUNCTION)**	
Do you feel that your child is performing all activities from before illness?	◯ YES<br>◯ NO<br>◯ N/A
Have you noticed any shortness of breath or taking more breaths than normal?	◯ YES<br>◯ NO<br>◯ N/A
Do you feel that your child's walking, jumping, going up stairs and running are at his baseline? (Gross motor skills)	◯ YES<br>◯ NO<br>◯ N/A
Do you feel that your child's fine motor skills are as they were? (coloring, fastening buttons, picking up legos)	◯ YES<br>◯ NO<br>◯ N/A
Do you feel their coordination is at their baseline? (playing sports, riding a bike, climbing on playground equipment)	◯ Yes<br>◯ No
Do you feel that his/her endurance for tasks are returned to normal? (running, swimming etc)	◯ YES<br>◯ NO<br>◯ N/A
Do you believe that your child has the physical ability to do all the activities that he/she like to do?	◯ YES<br>◯ NO<br>◯ N/A
Is he/she taking longer or more frequent naps?	◯ YES<br>◯ NO<br>◯ N/A
Is he/she sleeping through the night?	◯ YES<br>◯ NO<br>◯ N/A
How would you describe their overall energy level?	◯ Much better than before sepsis<br>◯ Little better than before sepsis<br>◯ About the same as before sepsis<br>◯ Little worse than before sepsis<br>◯ Much worse than before sepsis<br>◯ N/A

DEVELOPMENTAL (AGE SPECIFIC QUESTIONS)	
Is your child meeting their milestones? Examples are not sleeping in their bed, accidents of urine or stool, no longer sitting up.	◯ Yes<br>◯ No<br>(for infants and toddlers)
Have you noticed a loss of milestones since hospitalization? (school aged children)<br>Examples are fear of night time, separation anxiety from family or routine, not wanting to ride the bus.<br>For teenagers, examples are social isolation, lack of interest in driving, difficulty with video games.	◯ Yes<br>◯ No
bowel/urine elimination patterns	◯ Much better than before sepsis<br>◯ Little better than before sepsis<br>◯ About the same as before sepsis<br>◯ Little worse than before sepsis<br>◯ Much worse than before sepsis

THROBBING PAIN	
Has he/she complained of any new pain?	◯ Yes<br>◯ No
What is the severity of this pain?	______________________
Any new complaints of stomach pain or headache that is new?	◯ Yes<br>◯ No<br>(for young children and school aged children)
Do these occur when it is time for school or to go to the doctor's?	◯ Yes<br>◯ No

OVERALL CHANGES	
Has your child exhibited any new behaviors, or worsening of behaviors, that are concerning to you?	◯ Yes<br>◯ No<br>(specifically temper tantrums, hitting, tics, sleep issues, increase in seizure activity)
Have you noticed any changes since the illness that has not been covered by these questions? These could be changes in your child's physical health, thinking, emotional functioning, behavior, social skills or ability to do daily tasks.	◯ Yes<br>◯ No
Would you consider him/her the same as before the sepsis was identified?	◯ Yes<br>◯ No

EMOTIONS	
Does your child seem withdrawn or nervous?	◯ YES<br>◯ NO<br>◯ N/A
Have you noticed outbursts of anger or crying?	◯ YES<br>◯ NO<br>◯ N/A
If YES to Outbursts; what is the frequency and duration of these outbursts?	______________________________
Have you noticed impulsivity?I	◯ YES<br>◯ NO<br>◯ N/A
If YES to impulsivity; What is the frequency and duration of these impulse actions? (provide examples of interrupting and grabbing)	______________________________
Does he/she seem nervous or jittery?	◯ Much better than before sepsis<br>◯ Little better than before sepsis<br>◯ About the same as before sepsis<br>◯ Little worse than before sepsis<br>◯ Much worse than before sepsis<br>◯ N/A
Does he/she seem to be getting along with friends?	◯ Much better than before sepsis<br>◯ Little better than before sepsis<br>◯ About the same as before sepsis<br>◯ Little worse than before sepsis<br>◯ Much worse than before sepsis<br>◯ N/A<br>(school aged and older children question)
Does he/she seem hyperactive?	◯ Much better than before sepsis<br>◯ Little better than before sepsis<br>◯ About the same as before sepsis<br>◯ Little worse than before sepsis<br>◯ Much worse than before sepsis<br>◯ N/A
Does he/she startle easy or act jumpy?	◯ Much better than before sepsis<br>◯ Little better than before sepsis<br>◯ About the same as before sepsis<br>◯ Little worse than before sepsis<br>◯ Much worse than before sepsis<br>◯ N/A
What is the amount of time your child is happy?	◯ Much better than before sepsis<br>◯ Little better than before sepsis<br>◯ About the same as before sepsis<br>◯ Little worse than before sepsis<br>◯ Much worse than before sepsis<br>◯ N/A

What is the amount of time your child is sad or depressed?	◯ Much better than before sepsis<br>◯ Little better than before sepsis<br>◯ About the same as before sepsis<br>◯ Little worse than before sepsis<br>◯ Much worse than before sepsis<br>◯ N/A
